# Wide resection and reconstruction using the sandwich technique for primary undifferentiated pleomorphic sarcoma of the sternum: a case report

**DOI:** 10.1186/s44215-026-00251-8

**Published:** 2026-03-25

**Authors:** Masao Kobayashi, Satoshi Takenaka, Kei Shinyashiki, Ryu Kanzaki, Tomohiro Maniwa, Hidetoshi Satomi, Keiichiro Honma, Jiro Okami

**Affiliations:** 1 Department of General Thoracic Surgery, Osaka International Cancer Institute, 3-1-69, Otemae, Chuo-ku, Osaka, 541-8567 Japan; 2 Department of Orthopedic Surgery, Osaka International Cancer Institute, Osaka, Japan; 3 Department of Pathology, Osaka International Cancer Institute, Osaka, Japan

**Keywords:** Sternal sarcoma, Primary undifferentiated pleomorphic sarcoma, Extensive sternal resection, Chest wall reconstruction, Sandwich technique

## Abstract

**Background:**

Primary undifferentiated pleomorphic sarcoma (UPS) of the sternum is rare, and surgical resection remains the mainstay of treatment. Extensive sternal resection necessitates reconstruction to preserve respiratory function, protect mediastinal structures, and maintain upper limb support. Various reconstruction techniques have been described, but optimal methods for high-risk patients remain unclear. We report a case of total sternal resection reconstructed using a sandwich technique with polymethyl methacrylate (PMMA) and polytetrafluoroethylene (PTFE) sheets.

**Case presentation:**

A 79-year-old man with multiple comorbidities presented with a painful anterior chest wall mass. Imaging revealed a 3-cm localized sternal lesion, and biopsy confirmed UPS. Total sternal resection was performed, preserving the left pectoralis major muscle for flap coverage. A PMMA sheet sandwiched between PTFE sheets was anchored to the ribs, providing rigid yet lightweight chest wall support. A pedicled pectoralis major musculocutaneous flap covered the defect. Postoperative recovery was uneventful, with preserved pulmonary and upper limb function. Histopathology confirmed negative margins. At 8 months, a solitary left supraclavicular lymph node metastasis was treated with radiotherapy. The patient remains progression-free at 18 months.

**Conclusion:**

Total sternal resection followed by sandwich technique reconstruction can provide stable chest wall support while preserving respiratory and upper limb function, even in elderly patients with comorbidities. This case highlights atypical metastatic patterns of sternal sarcomas, emphasizing the need for careful follow-up. The sandwich technique is a safe and reproducible option for extensive sternal reconstruction in high-risk patients.

**Supplementary Information:**

The online version contains supplementary material available at 10.1186/s44215-026-00251-8.

## Background

Primary undifferentiated pleomorphic sarcoma (UPS) of the bone is rare, and surgical resection is considered curative [1]. When extensive sternal resection is performed, reconstruction is essential to preserve respiratory function, protect mediastinal structures, and maintain structural support for upper limb movement [2]. Various reconstruction techniques have been described, including musculocutaneous flaps, titanium plate systems, and modular multifunctional reconstruction plates [3]. However, optimal methods for elderly patients or those with comorbidities remain unclear. We report a case of primary UPS of the sternum managed with total sternal resection and reconstruction using the sandwich technique with polymethyl methacrylate (PMMA) and polytetrafluoroethylene (PTFE) sheets, highlighting technical considerations and clinical outcomes.

## Case presentation

**Fig. 1:**
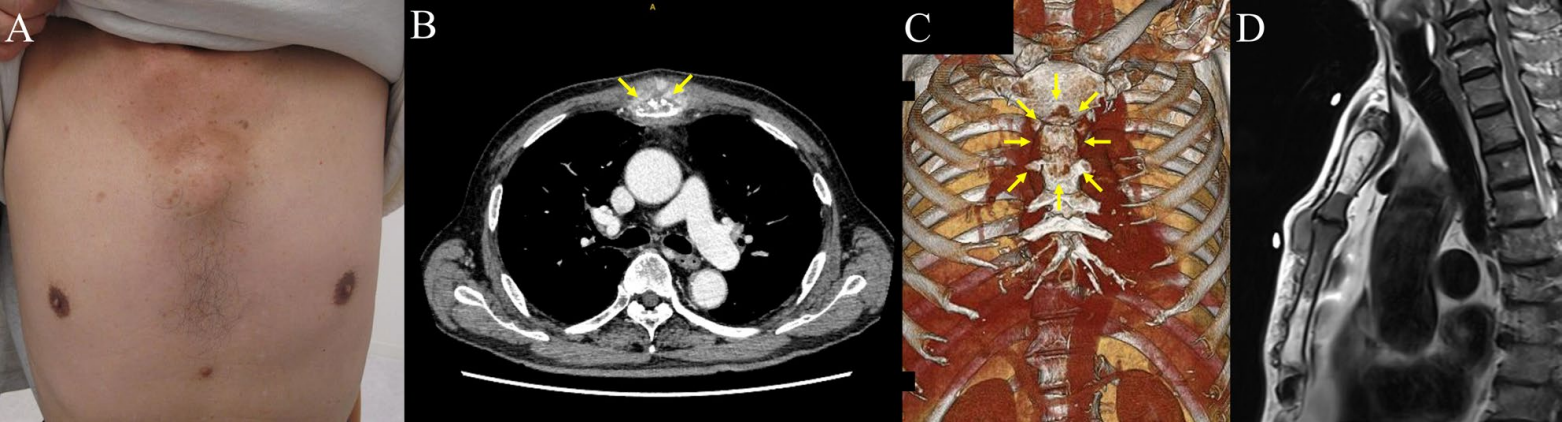
Preoperative gross findings and imaging of the sternal tumor. (**A**) Gross appearance. (**B**) CT image showing a 3-cm tumor in the sternum with osteolytic features. Arrows indicate the osteolytic areas. (**C**) Three-dimensional reconstructed CT image of the sternal tumor. Arrows indicate the tumor. (**D**) Magnetic resonance imaging revealing tumor invasion into both the manubrium and the body of the sternum. Abbreviations: CT, computed tomography.

A 79-year-old man with a history of cerebral infarction (on aspirin therapy), diabetes mellitus, hypertension, and hyperlipidemia presented with a painful anterior chest wall mass (Fig. 1A). Computed tomography revealed a 3-cm sternal lesion with osteolytic changes (Fig. 1B, C). Core needle biopsy revealed an undifferentiated malignant neoplasm. Positron emission tomography-computed tomography showed high fluorodeoxyglucose uptake (SUVmax 13.3) localized to the sternum. No other abnormal findings were detected. Magnetic resonance imaging demonstrated tumor invasion involving both the manubrium and sternal body (Fig. 1D). Considering possible intramedullary tumor spread, total sternal resection was selected to ensure adequate oncologic margins. Tumor markers were within normal limits. Based on these findings, the lesion was diagnosed as a localized primary malignant sternal tumor, and surgical resection was planned. The case was discussed at a multidisciplinary cancer board involving thoracic surgeons, orthopedic oncologists, medical oncologists, and radiologists. Multimodal treatment options, including perioperative chemotherapy, were considered; however, given the patient’s advanced age and multiple comorbidities, surgical management alone was selected.

**Fig. 2:**
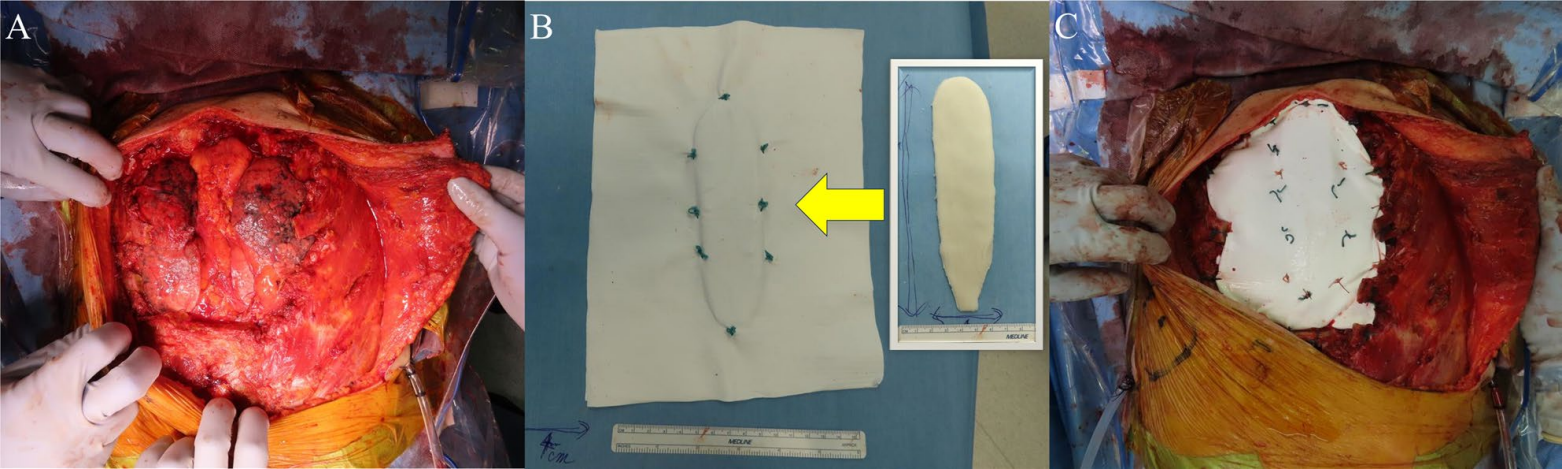
Intraoperative findings. (**A**) En bloc resection of the sternum, including the manubrium and upper sternal body. (**B**) Polymethyl methacrylate molded into the shape of the resected sternum and sandwiched between two polytetrafluoroethylene sheets. (**C**) The reconstructed prosthesis fixed to the residual rib stumps bilaterally.

Under general anesthesia, the patient was placed in the supine position. Skin and soft tissue were dissected with a surgical margin of at least 2 cm around the tumor. The left pectoralis major muscle was preserved in anticipation of flap reconstruction. After thoracoscopic evaluation, the third to fifth ribs were transected bilaterally. The sternum was divided at the caudal edge of the fifth intercostal space and elevated cranially. Bilateral second ribs, first ribs, and the sternoclavicular joints were sequentially divided, permitting en bloc resection of the sternum (Fig. 2A, Video 1). As lymph node involvement is uncommon in undifferentiated pleomorphic sarcoma, no elective nodal treatment had been performed. A prosthetic sternum was constructed using PMMA molded to a thickness of approximately 1 cm and sandwiched between two 1-mm PTFE sheets (Fig. 2B). The construct was anchored to the bilateral rib stumps using nonabsorbable 2 − 0 Ethibond sutures (Ethicon, Somerville, NJ, USA) (Fig. 2C). A large soft tissue defect was covered using a pedicled left pectoralis major musculocutaneous flap (Video 2). The patient was extubated in the operating room and had an uneventful postoperative course. He was discharged in stable condition on postoperative day 16.

**Fig. 3:**
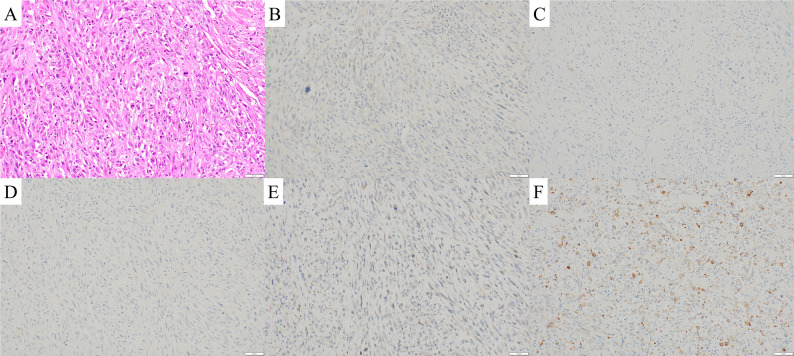
Histopathological and immunohistochemical findings of the sternal tumor. (**A**) Hematoxylin and eosin staining showing spindle cell proliferation. (**B**–**F**) Immunohistochemical staining revealing negativity for cytokeratin (**B**), S-100 protein (**C**), desmin (**D**), and MDM2 (**E**) and focal positivity for α-smooth muscle actin (**F**).

Histopathological examination revealed spindle cells. Immunohistochemistry was negative for cytokeratin, S-100, desmin, and MDM2 but partially positive for α-smooth muscle actin, confirming a diagnosis of UPS (Fig. 3A–F). Surgical margins were negative.

**Fig. 4:**
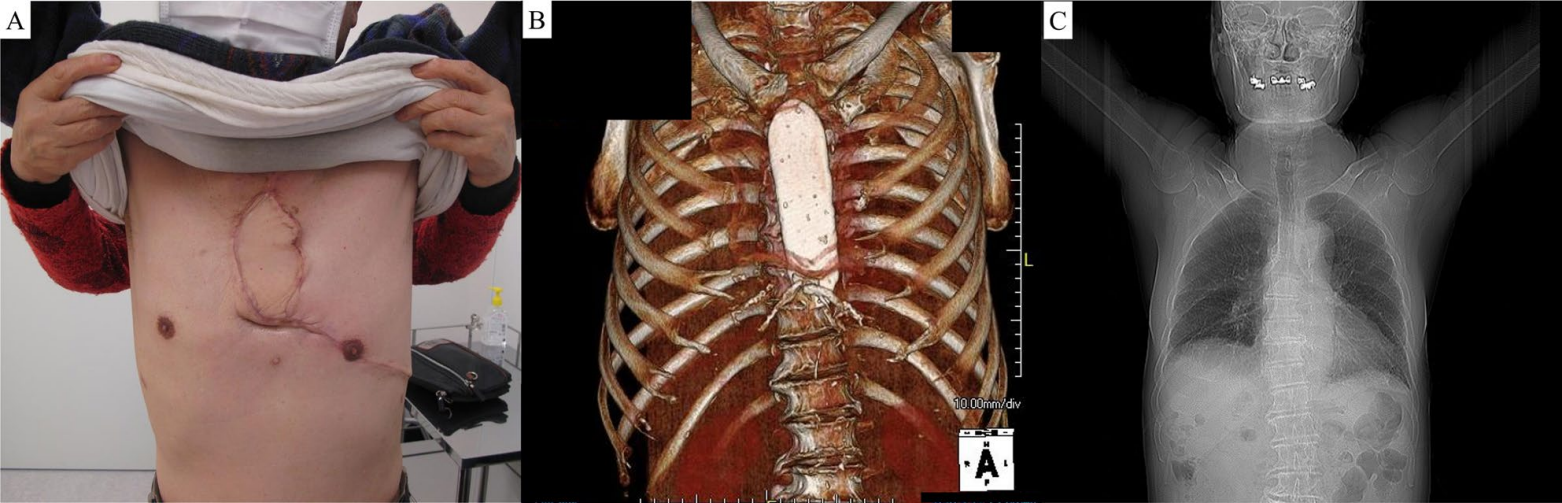
Postoperative course. (**A**) Gross appearance of the surgical site. (**B**) No displacement of the reconstructed sternum was observed. (**C**) Function of both upper limbs was preserved.

**Fig. 5:**
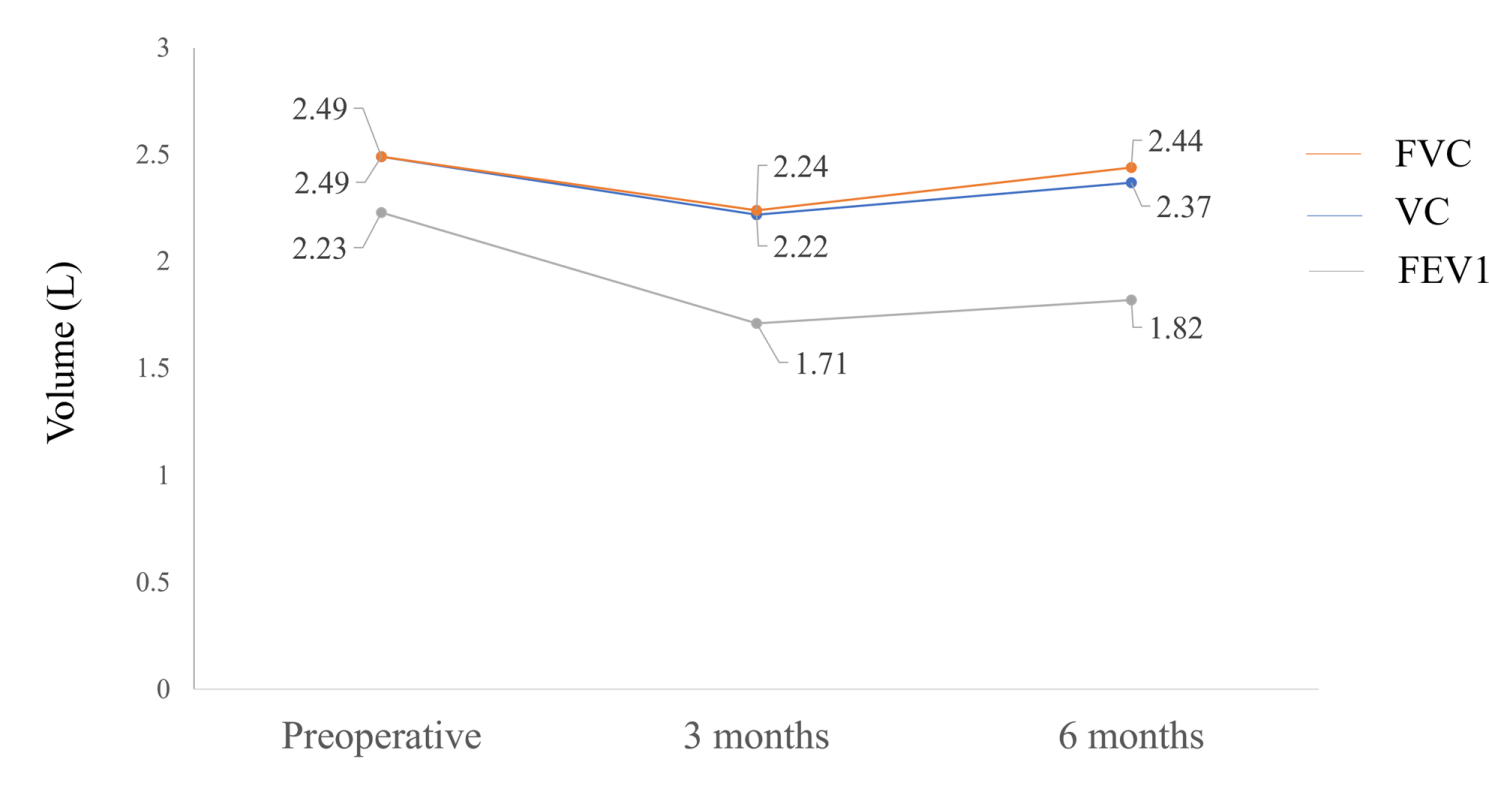
Changes in pulmonary function before and after surgery. Forced vital capacity (FVC), vital capacity (VC), and forced expiratory volume in one second (FEV1) were measured preoperatively and at 3 and 6 months postoperatively. All parameters decreased at 3 months. At 6 months, FVC and VC recovered to near preoperative levels, whereas FEV1 remained mildly decreased. The x-axis indicates Preoperative, 3 months, and 6 months, and the y-axis indicates Volume (L)

Postoperatively, there was no evidence of chest wall instability, and upper limb function was preserved (Fig. 4). The range of motion of both shoulders was 160° of flexion and 160° of abduction, and the patient’s activities of daily living were fully independent, indicating satisfactory functional recovery after reconstruction. Pulmonary function tests at 6 months showed only a mild decline in forced expiratory volume in one second (FEV1) (Fig. 5). At 8 months postoperatively, a solitary left supraclavicular lymph node metastasis was detected. The metastatic lesion was irradiated to a total dose of 60 Gy in 20 fractions while sparing the brachial plexus. The patient remains free of progression at 18 months.

## Discussion

UPS of the bone shows improved disease-specific survival with surgical resection combined with adjuvant chemotherapy [4]. However, owing to the patient’s advanced age and multiple comorbidities, chemotherapy was not administered.

UPS is considered a high-grade sarcoma, for which achieving an adequate surgical margin is critical for local control. According to the Clinical Practice Guidelines for Bone and Soft Tissue Tumors published by the Japan Society of Clinical Oncology, resection with approximately a 2-cm margin of normal tissue is associated with local recurrence rates of 7–13% in high-grade sarcomas and is recommended to maintain recurrence below 10% [5, 6]. Although evidence specific to chest wall or sternal sarcomas remains limited, the ≥ 2-cm soft-tissue margin adopted in the present case was considered oncologically appropriate based on these general principles. While pulmonary metastases are most common in UPS, lymph node involvement is rare [4]. Most reported cases originate from long bones; the sternal origin in this case, with its distinct lymphovascular anatomy, may explain the isolated supraclavicular lymph node metastasis. A large retrospective study of 5,064 patients from a Japanese bone sarcoma registry reported that regional lymph node metastasis (RLNM) occurred in 5.2% of patients with undifferentiated pleomorphic sarcoma, with isolated RLNM conferring survival outcomes equivalent to those with distant metastasis [7]. This demonstrates that, although uncommon, lymphatic spread in UPS can occur and may have important prognostic implications. As no other metastatic disease was identified, radiotherapy was used for local control in the present case. Given that isolated regional lymph node metastasis has been associated with survival outcomes comparable to distant metastasis, isolated lymph node involvement in this case may represent clinically significant disease and warrants careful long-term surveillance.

The sternum is essential for respiratory mechanics, mediastinal protection, and upper limb support. Reconstruction must balance durability, function, and complication risk. Musculocutaneous flaps alone have been associated with respiratory failure in some cases [8]. Various materials and prosthetic strategies have been proposed for sternal reconstruction, yet none fully satisfies all ideal requirements, and each approach has inherent limitations. Autologous bone grafting offers biological integration and potential new bone formation; however, its use remains constrained by donor availability and additional surgical trauma [9]. Prosthetic reconstruction has therefore become widely adopted. Titanium-based rigid reconstruction systems provide structural stability, facilitate contour restoration, and are associated with relatively low complication rates; however, they may be costly, can attenuate radiotherapy delivery, and remain susceptible to implant-related complications such as fluid collection or infection, and rigid plate constructs may additionally lead to adjacent rib fractures or implant failure [10, 11]. Three-dimensional printed prostheses allow precise anatomical matching of the defect, but their design and manufacturing are expensive and time-consuming, and their dependence on preoperative definition of resection extent may conflict with intraoperative oncologic decision-making based on surgical margins [12]. Each method has trade-offs in terms of operative time, cost, biomechanics, and invasiveness, highlighting the lack of a universally accepted “standard.” When determining the reconstructive strategy, the functional impact of the resection extent must also be considered. Although resection of the sternoclavicular joint was previously thought to compromise shoulder function, recent studies have demonstrated minimal functional deficits even after extensive resection [13]. In the present case, the patient’s advanced age, comorbidities, and ongoing antiplatelet therapy raised concerns regarding operative invasiveness and hardware-related complications. Although the sandwich technique has known potential drawbacks, including risks of infection and respiratory complications, as reported in previous series with large PMMA prostheses (infection 5–6%, respiratory failure 3–4%, rare cases of prosthesis dislocation or adjacent rib fracture), it was selected as a semi-rigid alternative because it allows intraoperative shaping to the defect, does not require complex fixation or extensive hardware implantation, and provides sufficient structural support to maintain respiratory mechanics while limiting procedural burden [14]. First described by McCormack et al. in 1981, this approach has been shown to achieve stable chest wall reconstruction with preserved respiratory function [15]. In our patient, it achieved adequate rigidity without prosthesis failure or rib fracture, and despite a mild reduction in FEV1, respiratory and functional outcomes remained satisfactory, demonstrating that the sandwich technique can be a safe and effective option in high-risk patients.

This case highlights the feasibility of the sandwich technique for sternal reconstruction in older patients with comorbidities. It also highlights the potential for unusual metastatic patterns in sternal sarcomas, supporting the need for further case accumulation and follow-up data.

## Supplementary Information

Supplementary Material 1.

Supplementary Material 2.

## Data Availability

The datasets used and/or analyzed during the current study are available from the corresponding author on reasonable request.

## Abbreviation

UPS: Undifferentiated pleomorphic sarcoma
PMMA: Polymethyl methacrylate
PTFE: Polytetrafluoroethylene
FEV1: Forced expiratory volume in one second
RLNM: Regional lymph node metastasis
